# Tonghua Liu: A life dedicated to clinical pathology

**DOI:** 10.1007/s13238-018-0601-0

**Published:** 2018-12-07

**Authors:** Lin Dong, Tanping Fu, Junyi Pang, Zhiyong Liang, Wenli Duan

**Affiliations:** Peking Union Medical College Hospital, Beijing, 100730 China

**Figure 1:**
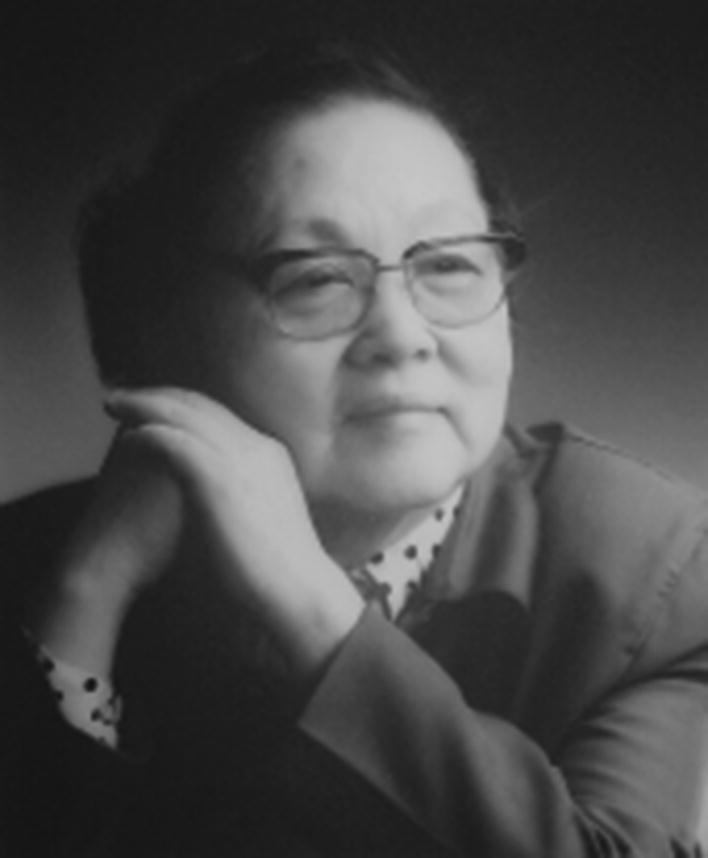
Professor Tonghua Liu (1929–2018)

Professor Tonghua Liu (刘彤华) was an academician of the Chinese Academy of Engineering, a renowned medical scientist, pathologist, medical educator and professor of the Pathology Department in Peking Union Medical College Hospital (PUMCH) (Fig. 1). She passed away from illness on July 8th, 2018 at PUMCH. She was 89 (PUMCH, 2018).

Prof. Liu was born on November 13th, 1929 in Wuxi, Jiangsu Province. She studied at the Medical School of Saint John’s University in Shanghai from 1947 to 1953 (Fang et al., 2009).

**Figure 2:**
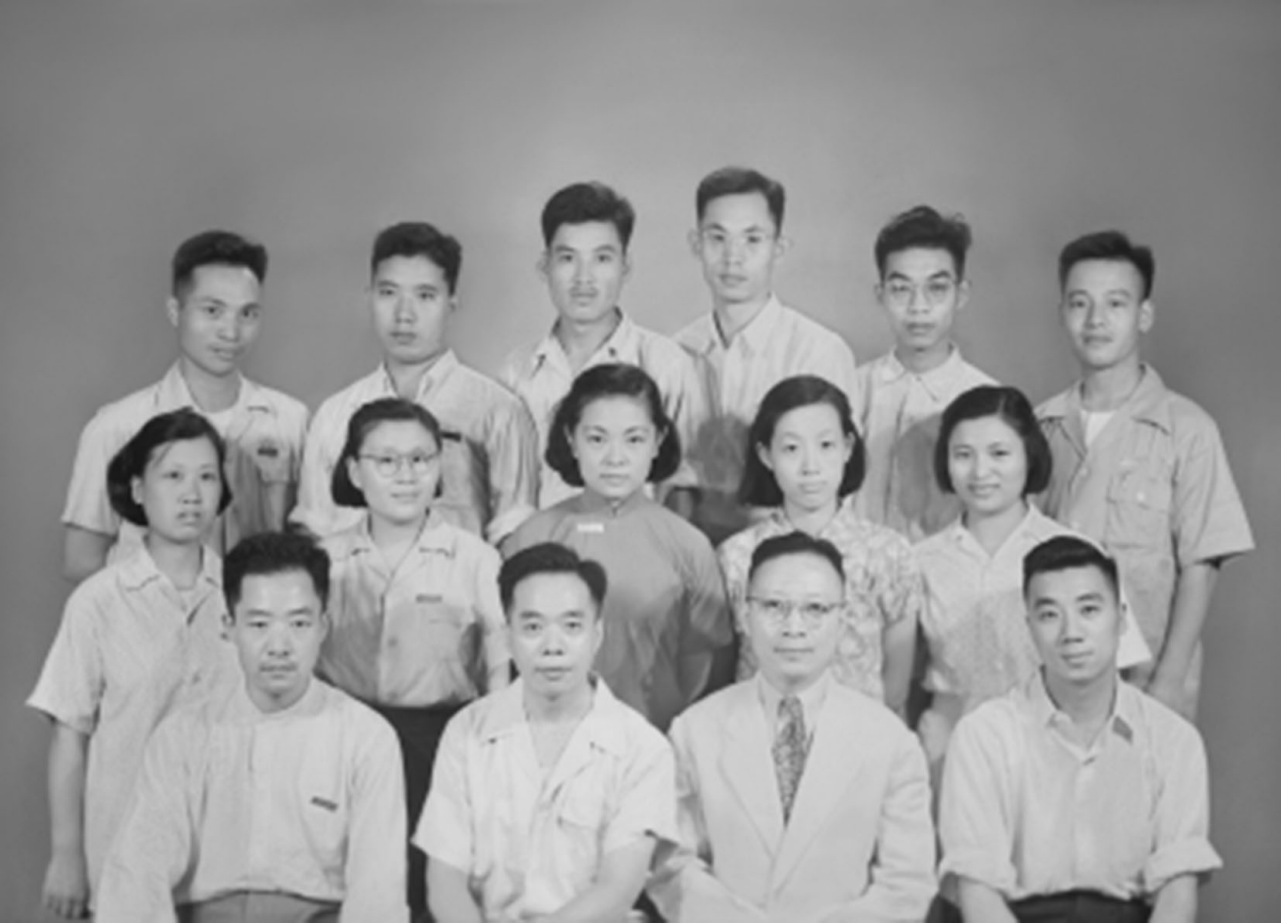
Faculty and Students from PUMC Pathological Senior Teacher Training Program in 1952, Tonghua Liu is the third in the second row, Prof. Zhengxiang Hu is the third in the front row

In the early 1950s, Professor Zhengxiang Hu (胡正祥), director of the Pathology Department of Peking Union Medical College (PUMC), was running a national Pathological Senior Teacher Training Program. Based on Liu’s educational background, she could only pursue basic medicine sciences. Despite this, Liu still dreamed of becoming a doctor. As a result she made the second best choice and applied for Prof. Hu’s program as a pathologist. This would allow her to remain close to both doctors and patients during clinical diagnosis. In 1952, Liu left Shanghai for Beijing to follow Prof. Hu for further studies (Fig. 2). Prof. Hu’s mentorship had a great influence on her. Prof. Hu’s teachings always stuck with Liu: “A scientific researcher shall completely immerse himself in science with no distractions.” (Duan et al., 2016).

In 1953, Liu graduated from Prof. Hu’s program. She became a teaching assistant at the 6th Army Medical University and was there from 1953–1954. In the following two years she worked for the 7th Army Medical University as a teaching assistant. Today the 6th and the 7th Army Medical Universities are now known as the 3rd Army Medical University (Fang et al., 2009).

In 1957, the Pathology Department of PUMC was merged with the Experimental Medicine Institute at the Chinese Academy of Medical Sciences (CAMS). From 1957 to 1969 Liu worked as both a teaching assistant at the Pathology Department of PUMC and a teaching assistant and assistant researcher at the Pathology Department at the Experimental Medicine Institute of CAMS. In 1969, the Experimental Medicine Institute moved to Jianyang, Sichuan Province. As a spouse of an army man, Liu could not separate from her husband. Thus she decided to stay in Beijing to found a separate Pathology Department in PUMCH (Duan et al., 2016).

The Pathology Department of PUMCH started from scratch. Almost all equipment and archives, including all autopsy files and gross specimen, had been moved to Jianyang. All that was left was a few office rooms and two technicians. Liu insisted that the 270,000 clinical pathological archives should be left to PUMCH, “Clinical pathological archives are critical to clinical studies. In order to make the best use of them, these files should be given to PUMCH.” A few years later, the previous autopsy archives were also transported back from Jianyang to Beijing (Liu, 2010). These documents were invaluable to the development of clinical pathology in PUMCH, and pathology in China in general. Because of Prof. Liu’s dedication, PUMCH now housed over 11 million pathological archives dating all the way back to 1916. The PUMCH pathological archive is a testament to Prof. Liu’s professionalism (Duan et al., 2016).

Prof. Liu was appointed deputy director of the Pathology Department of PUMCH in 1978 and then director in 1985 (PUMCH, 2018). Prof. Liu overcame great challenges so that the Pathology Department of PUMCH could grow and thrive. She voluntarily took over part of the technicians’ duties when the lab was severely understaffed during the initial years. She would come to the lab at 7 am every day to embed paraffin. This would allow the technicians to slice the paraffin and make section specimen at 8 am. After that, Liu would assess a great number of slices and generate reports. She always stayed late to review literature on difficult cases. Prof. Liu’s diligence has become a tradition in the Pathology Department of PUMCH (Duan et al., 2016).

Prof. Liu signed her name on almost 1 million clinical pathology reports during her 65-year career. She always gave definitive diagnosis, embodying her capable and decisive temperament. Prof. Liu was famous for diagnosing rare and difficult cases, especially in the field of lymph nodes, digestive and endocrine diseases. Her diagnosis was held as the “golden standard” across the country. In the 1990s, Prof. Liu was consulted and made a diagnosis of cervical lymph node metastatic squamous cell carcinoma. After receiving Prof. Liu’s report, doctors examined the patient carefully, seeking to identify the primary tumor lesion. However, they found nothing and doubted Prof. Liu’s diagnosis. The doctors requested Prof. Liu review the case again. After re-reviewing the case, Prof. Liu’s diagnosis remained unchanged but she added 4 words to her report, “examine patient’s oral cavity.” At last, doctors were able to find an unconspicuous primary tumor lesion on the patient’s gum. When it came to diagnosis, her suggestion had ascended from science to art (Duan et al., 2016).

Prof. Liu always believed in drawing on the PUMCH tradition of combining basic sciences with clinical research. In the 1980s and 1990s, her team achieved a series of innovative breakthroughs on molecular biology and molecular genetics of pancreatic carcinoma and pancreatic neuroendocrine tumours (PNET). Her team’s study on “Pancreatic Dead Carcinoma’s Ring Invasion into Intrapancreatic Bile Duct Wall” won the Second Prize of Science and Technology Progress Award of the National Health Commission in 1985 (Liu et al., 1983). Her team won another Second Prize of Science and Technology Progress Award of the National Health Commission in 1993 for their study of “Molecular Biology of Human Pancreatic Carcinoma Cell and Reversal of its Malignant Phenotype by Antisense Gene Regulation” (Chen et al., 1993). Another study on “Antisense Gene Regulation Reverse Malignant Phenotype of Human Pancreatic Carcinoma Cell” won the Second Prize of the National Science and Technology Progress Award in 1995 (Liu et al., 1995). In 1999, Prof. Liu was elected the academician of the Chinese Academy of Engineering for her outstanding achievements in pathogenesis and gene therapeutics of pancreatic carcinoma. She was the first pathologist to be elected as an academician of the Chinese Academy of Engineering (PUMCH, 2018).

**Figure 3:**
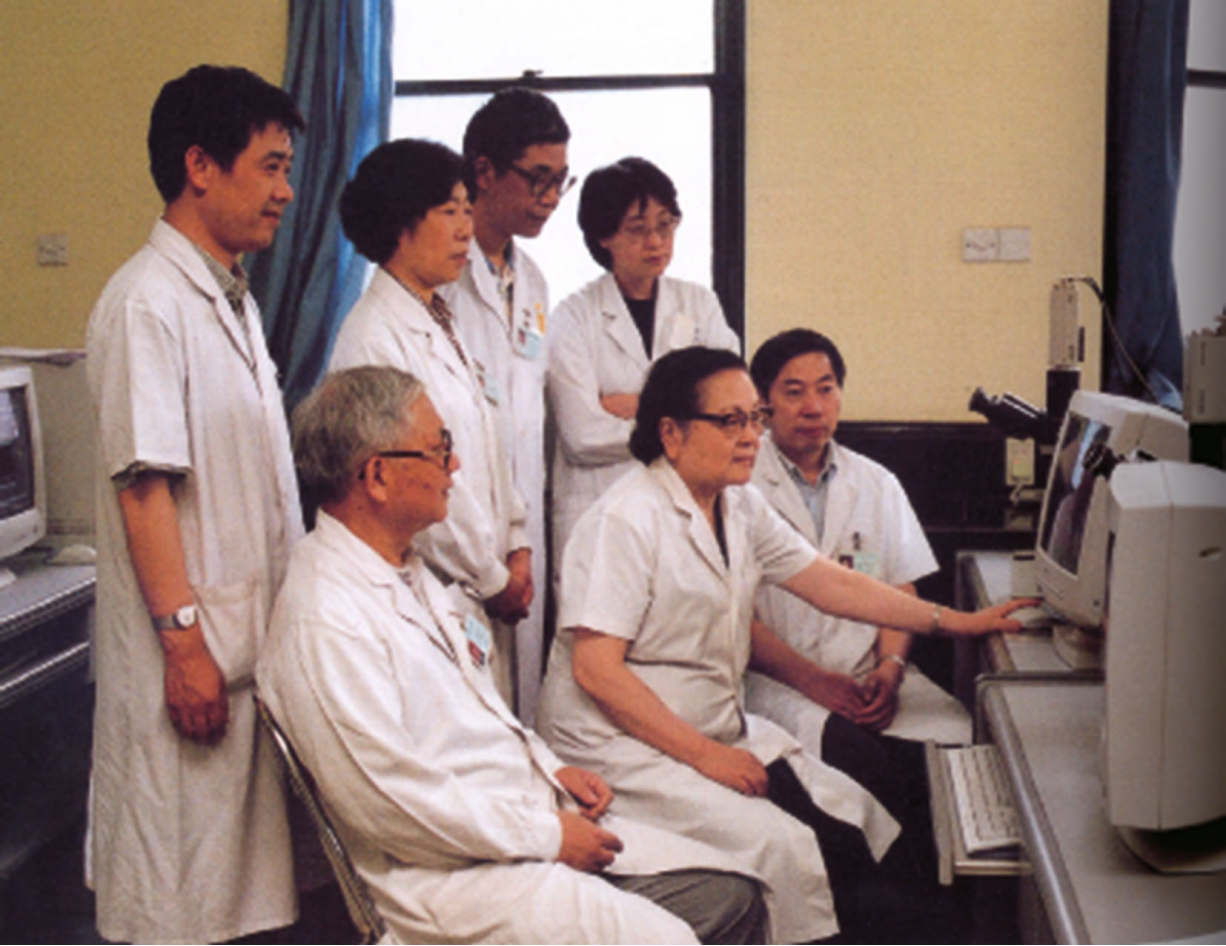
Tonghua Liu and her colleagues from Pathology Department of PUMCH in 2001

Prof. Liu held a great vision for scientific development. In the late 1990s, Prof. Liu sensed the prospects of molecular biology. She sent young pathologists and technicians abroad to study in this field. By combing traditional pathomorphology with molecular biology, many new technologies were applied to clinical diagnosis. Entering the 21st century, Prof. Liu foresaw the future of biological targeted therapy (Fig. 3). She put forward the notion that targeted therapy needed targeted diagnosis (Liu, 2008). In 2003 she established the first molecular genetics pathology lab in China. Many renowned international pharmaceutical companies sought for cooperation with her. Global clinical trials for new antineoplastic targeted drugs were undertaken in PUMCH. Prof. Liu led her team digging into the targeted diagnosis of breast cancer, gastric cancer, colorectal cancer, lung cancer and many other diseases. These efforts expanded the application of clinical pathology, improving pathologists’ importance in the multidisplinary team, bringing hope to the increasing number of tumor patients in China (Fang et al., 2009).

Prof. Liu was an executive editor of the third and fourth editorial board of the *Chinese Journal of Pathology*, and the honorary editor-in-chief of its eighth editorial board (Huo et al., 2009). She was also the honorary editor-in-chief of *Chinese Journal of Diagnostic Pathology* (Liu, 2014) and a member of the standing committee of the Society of Pathology, Chinese Medical Association. She published more than 400 theses and was chief editor of four academic books. *Diagnostic Pathology* was issued under her general editorship in 1994. It won the National Excellent Science and technology Book Award in 1995. This book has since become an essential reference for all Chinese clinical pathologists (PUMCH, 2018). In memorial of Prof. Liu’s contribution, the fourth edition of the book was renamed to *Liu Tonghua Diagnostic Pathology* (Dong & Pang, 2018).

Prof. Liu also won many other titles and awards including the National Distinguished Teacher & Beijing Distinguished Teacher award in 1995, Expert of Outstanding Contribution by National Health Commission in 1998, Capital Labor Medal in 2003, Distinguished Worker from the China Association for Science and Technology, Beijing March 8 Red Flag Bearer, Beijing Patriotic and Contributing Model, Renowned Doctor of PUMCH, Distinguished Contribution Award and Distinguished Expert from PUMCH etc. (PUMCH, 2018).

**Figure 4:**
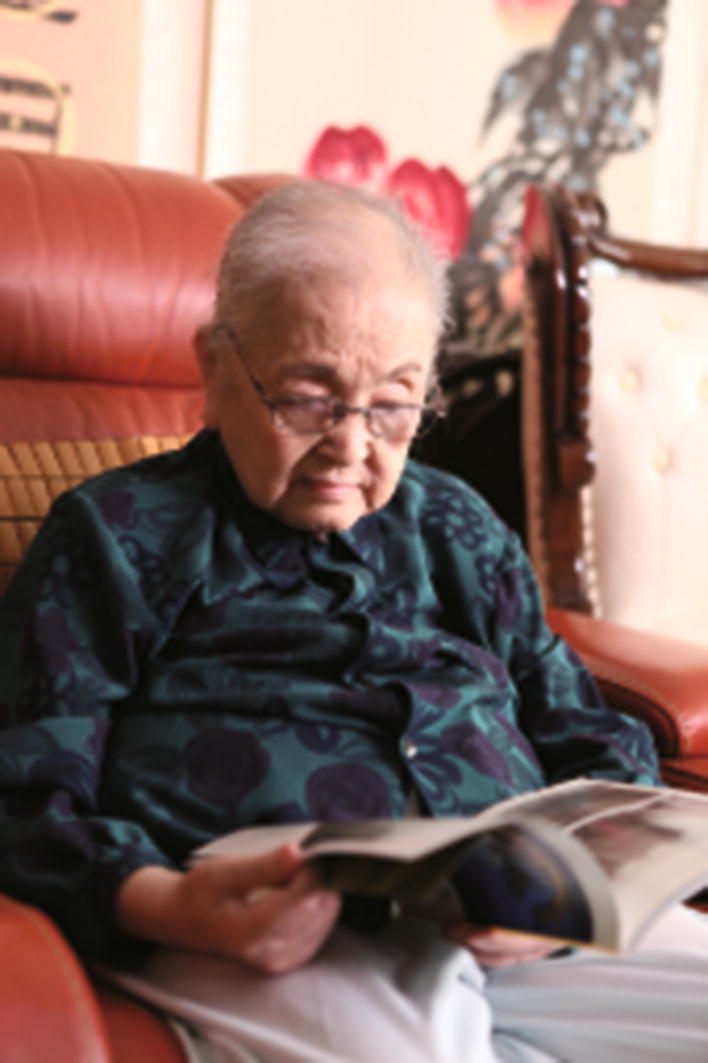
Reading magazine at her house on Jun 6th, 2018, Tonghua Liu was interviewed by PUMCH’s Oral History Program. This is her last picture. She passed away one month later

Prof. Liu spent her whole life in the company of microscopes and magnifying lens, making significant contribution to China’s pathology undertakings and education, setting an outstanding example for later generations (Fig. 4) (Duan et al., 2016).
